# Editorial: Interaction of Gas Messengers With Mitochondria in Health and Disease

**DOI:** 10.3389/fmed.2018.00259

**Published:** 2018-09-26

**Authors:** Andrey V. Kozlov, Mihaly Boros

**Affiliations:** ^1^Ludwig Boltzmann Institute for Experimental and Clinical Traumatology in AUVA Research Center, Vienna, Austria; ^2^Faculty of Medicine, Institute of Surgical Research University of Szeged, Szeged, Hungary

**Keywords:** nitric oxide, carbone monoxide distribution, hydrogen sulfide (H 2S), methane, mitochondria

Since a decade three gases nitric oxide (NO), carbon monoxide (CO), and hydrogen sulfide (H_2_S) are recognized as being produced by eukaryotic cells to serve as messengers in intracellular signaling pathways. More recently methane (CH_4_) was recognized as the 4th gas messenger. All four gases not only act in a defined range of physiological processes, but also contribute to the development of a number of diseases. All four gas messengers exert similar biological effects and have similar target structures. One of the most important target structures for all four gaseous mediators are mitochondria. Mitochondria are relatively autonomous intracellular organelle in eukaryotic cells. They regulate many essential intracellular processes such as energy supply, reactive oxygen species mediated signaling, oxidative stress, programmed cell death, turnover of Ca^2+^, several metabolic pathways related to lipid and protein metabolism. It is less clear how mitochondrial functions are regulated from the up-stream side and how up-stream regulatory signaling pathways are transformed by mitochondria into down-stream physiological and pathological manifestations of health and diseases. Endogenous gaseous messengers, NO, CO, H_2_S, CH_4_ are known to have a high affinity to different segments of mitochondria and are able to modulate efficiently mitochondrial functions. This suggests that endogenous gas messengers may orchestrate a reach pallet of pathways mediated by mitochondria.

This issue aims at collecting scientific reports describing novel aspects of interaction between gas messengers and mitochondria in terms of molecular mechanisms and the impact of combined effects of two or more gas messengers on mitochondria and intracellular pathways regulated by mitochondria. The issue contains reviews as well as original research articles.

In their review, Hartmann et al. have presented an overview on deleterious and beneficial effects of NO, CO, and H_2_S mediated by mitochondria. The authors summarize the current knowledge on the modulation of mitochondrial function by these three gas transmitters, with particular focus on the potential induction of suspended animation. Collectively the data analyzed by the authors suggest that gas messengers are able to induce a hypometabolic state, which is capable to hibernate the whole organism or isolated organs. They discuss potential application of this phenomenon for critical care and transplant medicine.

Duvigneau and Kozlov in their review considered conditions facilitating the interaction of NO and CO with mitochondria. They suggested four key factors, which determine the result of this interaction, including: (i) local concentration of NO and/or CO, (ii) tissue oxygenation, (iii) redox status of the cell in terms of facilitating or inhibiting reactive oxygen species (ROS) formation, and (iv) the degree of tissue acidosis. The combination of these four factors in specific physiological and pathological situations defines whether effects of NO and CO are beneficial or deleterious as well as magnitudes of these effects.

Mészáros et al. reported recent experimental data on biological functions of CH_4_. Recent studies have unambiguously confirmed CH_4_ bioactivity in various *in vitro* and *in vivo* experimental models. They provide strong evidence that CH_4_ affects mitochondrial physiology. The authors describe CH_4_ mediated signaling pathways including anti-apoptotic effects of CH_4_, which is mediated by mitochondria as well as induction of Nrf2/ARE-mediated activation of antioxidant system and detoxifying enzymes. The later attenuates the excessive production of ROS and preserves mitochondrial function. Moreover, recent publication of the same group shows that CH_4_ is able to influence the levels of NO, particular via regulatory effect on the nitrite reductase activity (1).

Dikalov et al. address the question regarding the interaction between gas messengers and reactive oxygen species (ROS) with the focus on the reactions involving NO. It is known that physiological generation of NO reduces oxidative stress and inflammation, however, the precise mechanism of this “antioxidant” effect of nitric oxide is not completely understood. The authors show that NO attenuates production of mitochondrial superoxide by post-translational modifications of specific proteins via nitrosylation of their cysteine residues and formation of protein dinitrosyl iron complexes. They suggest that the reduction of NO levels is associated with endothelial dysfunction due to the activation of mitochondria mediated oxidative stress.

Osipov et al. report the evidence suggesting that protein nitrosyl complexes are a potential source of specific free NO pool, which controls critical metabolic processes in the cell. They show that the release of NO in this pool can be controlled by photolytic decay of iron-sulfur protein nitrosyl complexes, enabling local control on the mitochondrial respiration. The authors discuss possible therapeutic applications of this NO pool.

Müllebner et al. reported experimental data on the contribution of NO and CO to the phagocytic activity of macrophages. They show that enzymatic complexes generating NO and CO contribute to the bactericidal activity of macrophages independently controlling different signaling pathways. They have shown that hemin, the substrate of heme oxygenase, rather than its products (biliverdin, iron ions, CO) affect the rate of phagocytosis, but has no effect on NADPH-oxidase activity. In contrast, NO does not affect the rate of phagocytosis, but modulates bactericidal activity of NADPH-oxidase.

Boyko et al. report a preclinical study on the pathological role of gas messengers in the central nervous system. Their experiments demonstrate that decreased NO• production from both L-Arginine and L-homoarginine occurs in the cortex several weeks after spinal cord injury and this decrease impairs antioxidative capacity of central nervous system.

Shvedova et al. report on the impact of gaseous messengers in ischemic preconditioning in the heart. The authors present the data suggesting that mitochondrial ATP-sensitive and Ca^2+^-activated K^+^ channels are involved in a specific NO-mediated signaling pathway in the heart. This pathway plays a key role in the ischemic preconditioning of the heart. Moreover, they suggest that Connexin 43 located in the inner mitochondrial membrane plays a central role in this mechanism.

In summary, this issue provides evidences that mitochondria mediated effects of gaseous messengers are an important regulator of intracellular processes, which has a strong therapeutic potential. Many questions regarding exact mechanisms of interaction between mitochondria and gas messengers are still opened and require further intensive studies.

## Author contributions

All authors listed have made a substantial, direct and intellectual contribution to the work, and approved it for publication.

### Conflict of interest statement

The authors declare that the research was conducted in the absence of any commercial or financial relationships that could be construed as a potential conflict of interest.
